# Barriers and Facilitators to Leisure Physical Activity in Children: A Qualitative Approach Using the Socio-Ecological Model

**DOI:** 10.3390/ijerph17093033

**Published:** 2020-04-27

**Authors:** María Martínez-Andrés, Raquel Bartolomé-Gutiérrez, Beatriz Rodríguez-Martín, María Jesús Pardo-Guijarro, Miriam Garrido-Miguel, Vicente Martínez-Vizcaíno

**Affiliations:** 1 Faculty of Nursing, University of Castilla-La Mancha, 02071 Albacete, Spain; Raquel.Bartolome@uclm.es (R.B.-G.);; 2 Social and Health Care Research Center, University of Castilla-La Mancha, 16071 Cuenca, Spain; Beatriz.RMartin@uclm.es (B.R.-M.); MariaJesus.Pardo@uclm.es (M.J.P.-G.); Vicente.Martinez@uclm.es (V.M.-V.); 3 Department of Psychology, University of Castilla-La Mancha, 02071 Albacete, Spain; 4 Faculty of Health Sciences, University of Castilla-La Mancha, 45600 Talavera de la Reina, Toledo, Spain; 5 Faculty of Education, University of Castilla-La Mancha, 16071 Cuenca, Spain

**Keywords:** focus groups, children, preadolescents, family adjustment, physical activity, qualitative research

## Abstract

Despite the benefits of engaging in physical activity during their leisure time, children do not meet the recommendations on physical activity. Following the socio-ecological model as a theoretical framework, the aim of this study was to determine the barriers and facilitators that influence physical activity participation in children’s leisure time. Data collection was conducted through focus groups and individual drawings in a sample of 98 eight- to eleven-year-olds from six schools in Cuenca (Spain). Following the socio-ecological model, individual characteristics (age and sex), as well as the microsystem (parents and friends), mesosystem (timing and out-of-school schedule) and exosystem (safety and weather) influence physical activity participation. The relationships between these levels of the socio-ecological model reveal that opportunities for leisure physical activity are determined by children’s schedules. This schedule is negotiated by the family and is influenced by parents’ worries and necessities. This is the main barrier to physical activity participation due to the creation of more restrictive, sedentary schedules, especially for girls. Our results show the elements required to develop successful strategies to increase physical activity opportunities, namely, focusing on giving children the opportunity to choose activities, raising parents’ awareness of the importance of physical activity and improving the perceived safety of parks, taking into consideration the gender perspective.

## 1. Introduction

Physical activity during childhood and adolescence has been associated with physical, psychological and social benefits [1,2]. Physical activity is defined “as any bodily movement produced by skeletal muscles that results in energy expenditure. […] Physical activity in daily life can be categorized into occupations, sport, conditioning, household, or other activities” [3]. 

Currently, it is recommended that children and adolescents accumulate at least 60 min of moderate-to-vigorous intensity physical activity on most days of the week [4], and so obtain health and psychosocial benefits that may sustain into adulthood [5,6]. However, the prevalence of individuals of these ages who do not meet these recommendations is growing at an alarming rate and is higher in girls [7,8,9]. Therefore, increasing the amount of time children and youngers dedicate to physical activity is an urgent concern in many countries.

Leisure time, along with the school, has been identified as a perfect moment to practice physical activity and support healthy behaviors, through active play and sports [10,11]. In addition, it is an opportunity for children to choose the activities they engage in (whether active or not) [12,13,14]. Previous studies focusing on leisure time have concluded that physical activity and its practice are affected by individual (age and fitness), psychological (preferences and satisfaction), sociocultural (gender, family and friends) and infrastructural influences (equipment, security and proximity) [13,15,16,17,18,19].

Furthermore, reviews and meta-analyses have concluded that the outcomes of programs to promote physical activity after school time are modest and inconsistent [20,21]. Moreover, to the best of our knowledge, most of the research focused on measuring the amount of physical activity [7,20,22], as well as identifying the barriers or facilitators to physical activity participation, use questionnaires [13,17,18,19,23,24,25,26] or are based on the perspective of adults (parents and teachers) and adolescents, while fewer directly center on children’s own perceptions [27,28,29,30].

Despite the importance of leisure time to create healthy-life patterns for children, few qualitative studies have attempted to understand the factors affecting physical activity practice from the perspective of children [27,28,29,30]. Recent studies have shown that children’s and adolescents’ perception of their options during their leisure time have an impact on their physical activity practice [13,19,25,31]. In addition, discrepancies have been found between the preferences of children and parents [27] as well as the influence of parents on their children’s physical activity participation. Two models have been identified: one promoting participation in physical activity, and the other restricting such participation [32,33]. However, most of these studies failed to analyze gender differences, despite their importance [27,34,35,36].

Engaging physical activity is, therefore, a complex matter because exercise-related behaviors are influenced by multiple factors at different levels [37,38,39]. Bronfenbrenner’s socio-ecological model [40] is a key approach in this field because it integrates both factors and dimensions that impact this behavior. This is especially interesting in our study given that developmental models help understand development as a social process, as a result of the interaction between people and their environment. The socio-ecological model proposes four levels: individual characteristics (sex, age and preference), microsystem (family, teachers and friends), mesosystem (neighborhood, school and physical family) and exosystem (economics, culture and politics) [41]. This developmental and social perspective is fundamental when designing interventions and public policies focused on the promotion of a successful and effective healthy lifestyle [32,41]. Across these levels, family and its role in socialization play a fundamental role in the motivation of children in the practice of physical activity and its continuance during their adolescence [42,43,44].

In this sense, the promotion of physical activity requires a physical and psychosocial environment to help promote facilitators and reduce barriers. To the best of our knowledge, a number of studies, mainly quantitative ones, have addressed children’s perception of barriers and facilitators when they participate in physical activity during their leisure in southern Europe, especially in Spain, and in relation to family dynamics [13,17,18,19]. The results of two recent studies focused on how children and adolescents presented barriers (gender inequalities, time constraints and lack of safety) and facilitators (friends and time sports) during their leisure time in Spain [45,46], one of which adopted a qualitative approach. However, neither focused solely on the child population. 

In light of the above, the aim of this study was to identify the barriers and facilitators that influence the practice of physical activity in children’s leisure time, using the socio-ecological model as a theoretical framework. Thus, our research question was the following: What factors act as barriers or facilitators for children during their leisure time?

## 2. Methods

To respond to the research question, we considered a qualitative descriptive study, which was the most appropriate method as it helps to identify the most significant factors or processes within a theoretical model of a phenomenon, as is our case, and drawing on a naturalistic and flexible approach [47,48]. The aim of our study was to determine the factors that influence physical activity through children’s discourse on their daily activities, within their own context. Children, due to their stage of cognitive development, tend to focus on very specific facts. Furthermore, a qualitative description allowed us to interpret the meaning children give to these specific facts in relation to physical activity.

### 2.1. Participants

This qualitative study is part of the MOVI-2 project [49]. MOVI-2 is a recreational and non-competitive (i.e., handball, hockey, steal the flingsock and dance) after-school physical activity intervention involving children from 20 schools in the province of Cuenca (Spain) (www.movidavida.org). The participants were 8- to 11-year-old schoolchildren in the 4th and 5th grades of primary education. 

The present qualitative study used a convenience sample and was conducted between January and April of 2011. The final subsample was formed by 98 schoolchildren, belonging to 6 schools from the MOVI-2 project. The researchers finished data collection when saturation point was reached [50,51].

The inclusion criteria were both sexes, belonging to a different socio-economic status and from schools located in urban and rural settings (participating schools from MOVI-2) (Table 1). The qualitative part of the main MOVI-2 study had three research questions seeking to identify barriers and facilitators to the practice of physical activity in different contexts. 

Meetings were held with parents to inform them about the aims and methods of the MOVI-2 study, and to request participation in the intervention. The qualitative study, sessions and films required separate informed consent. All the children agreed to participate, except for two girls, whose parents did not wish them to be filmed. Data collection was completed when participants provided no further new information and the saturation point was reached [50,51].

### 2.2. Data Collection

A descriptive qualitative study was designed by combining two data-collection techniques: (i) analysis of the children’s drawings of their environment [52] and (ii) focus groups [53]. Each session was conducted by two researchers (M.M.A. and B.R.M.), one of whom acted as a moderator and the other as an observer. Twenty-two sessions involving four or five participants were conducted (18 mixed sessions and 4 female-only sessions), with each lasting an average of 40 min. All sessions took place in the schools and were recorded on audio and video.

All sessions began by requesting the participants to complete a personal drawing, consisting of a map with the places they usually go during the week [54]. Children were given 20 min to complete the drawing. The use of drawings helped the researchers to find out more about the main places they usually went, but also helped the participants to express their opinions during the focus groups. After completing the drawings, the children were divided in focus groups supported by a script (Table 2) based on the different levels of the sociological model and the personal drawings.

Nonetheless, due to the participants being children aged between 8 and 11, answers were occasionally very short. In order to obtain enough qualitative descriptions that were sufficiently rich, researchers used follow-up questions, thus enabling the participants to describe their experiences and points of view. A moderator identified the main topics in the participants’ comments and asked them further questions.

### 2.3. Data Analysis

We used qualitative content analysis, which is a flexible and dynamic strategy to analyze verbal and visual information [55]. Thus, it is possible to combine both inductive and deductive analysis to describe and interpret the information [56,57]. This is appropriate when describing the phenomenon to be conceptualized in a model or applying it in an existing one, such as the socio-ecological model. Using this model, we followed a conceptual framework to code the barriers and facilitators verbalized by the children. We then used an inductive analysis to reveal the latent themes.

Transcripts from the focus group recordings were sorted and organized accordingly, and three qualitative methodology experts (M.M.A., B.M.R. and R.B.G.) analyzed these data following a 3-step approach: (1) the data were divided into codes (matrix of barriers and facilitators in each system); (2) these codes were grouped into categories and subcategories; and (3) the categories were abstracted to latent themes and the relationship between systems. This process started with an analysis of all the information extracted from drawings and focus groups, which was organized in categories and themes. The information was then re-analyzed, taking into consideration the age and gender of the participants. If there were discrepancies for any participants, the speaker’s discursive contributions were not taken into account. The process was a continuous comparative method in which codes, categories and themes were discussed with the research team until all the members agreed.

The F4 Software tool was used to transcribe the focus groups and Atlast.TI 5.0 was used to process the data (drawings and text analysis). Drawings and focus groups were used to triangulate the data [51,58]. In this way, following the socio-ecological model, the researchers analyzed the correspondences between the oral discourses and the drawings.

### 2.4. Ethical Issue

All parents or guardians gave their informed consent for their children’s participation in the study. The study was conducted in accordance with the Declaration of Helsinki, and the protocol was approved by the Ethics Committee of the *Virgen de la Luz* Hospital in Cuenca, Spain (Protocol ID 2008PI0708).

### 2.5. Quality Criteria

Several strategies were used to guarantee a rigorous analytical approach. The credibility and reliability of the findings were enhanced through the selection of the participants: urban and rural settings, both sexes, different school year and different ages. The drawings were used to encourage children to express what best described their daily life based on the places they usually went, thus generating a relaxed atmosphere which encouraged them to talk. In order to explore the adaptability and suitability of the techniques to the characteristics of the participants, four trial sessions were carried out in a meeting room at the University of Castilla-La Mancha. In these sessions, the researchers checked different kinds of drawings (group or individual dynamic), different kinds of groups (only boys, only girls and mixed) and also refined the script with everyday words suitable for children. Finally, the coding was analyzed by three separate researchers [50,58]. After each level of coding had been completed, the researchers held a discussion to identify similarities and discrepancies.

The team worked reflexively, especially in the inductive part of the analysis, trying to control how prior knowledge about the phenomenon could influence the interpretation of the data. Not only did the researchers discuss agreement on codes, categories and themes, the team also looked at the possible influence of their professional or personal opinions and knowledge. The team comprised researchers with different backgrounds, which favored reflection on the data analysis. The search for agreement between the three researchers was not dominated by the search for a “lowest common denominator”, but by reflective discussion on interpretation [48,59].

## 3. Results

**Figure 1:**
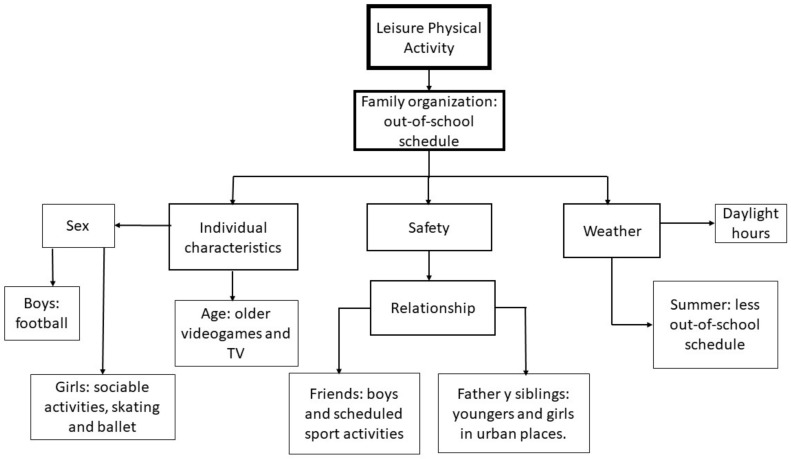
Code diagram.

The results were obtained and organized following the socio-ecological model, which showed the multiple factors involved in the children’s leisure physical activity. We found factors, barriers and facilitators, at the following levels of the socio-ecological model: individual characteristics, microsystem, mesosystem and exosystem (Figure 1 and Table 3).

### 3.1. Individual Characteristics of the Participants

Both boys and girls showed clear differences in personal tastes regarding the kind of activities performed. The boys chose games of vigorous-to-moderate intensity physical activity, mainly football.

*“On Wednesdays and Fridays I go to football training” (IP56.Boy, rural environment).*

*“When I’m not training, I also play football” (IP427.Boy, urban environment).*

The girls preferred socialization activities, such as pretend play, handicrafts or relational activities. Very few opted for sports or active games, as they did not find them enjoyable and said they involved making an effort. They typically chose activities such as ballet or skating.

*“I do craft-work and take skating lessons” (IP415. Girl, urban environment).*

*“I play teachers with my sister” (IP129. Girl, rural environment).*

*“I used to go to swimming and rhythmic gymnastics classes but gave up because I didn’t like them, it was a lot of hard work” (IP529. Girl, urban environment).*

*“I stopped going to ballet and swimming because I got bored and it was dull” (IP482. Girl, urban environment).*

Moreover, the older participants, both boys and girls, preferred to play videogames and watch TV in their leisure time, rather than play active games.

*“I love watching TV” (IP539. Boy, urban environment).*

*“I prefer playing Wii^®^” (IP118. Girl, rural environment).*

### 3.2. Microsystem: Parents, Siblings and Friends

Most of the participants shared the games with their fathers and siblings, but not with their mothers. Moreover, some of the children only engaged in leisure activities with their parents during the holidays.

*“I ride my bike or scooter with my brother and my father” (IP717. Boy, urban environment).*

*“I don’t do many things with my parents” (IP694. Girl, urban environment).*

*“No, I used to go to flamenco lessons, but I had to stop because my mum and dad were working and couldn’t drive me there” (IP516. Girl, rural environment).*

*“In the summertime, my parents and I go to the beach. But in M (village), I don’t spend much time with them” (IP609. Boy, rural, environment).*

As they grew older and were allowed to go out alone, the children stopped sharing games and leisure activities with their families and started engaging in activities only with their friends. Scheduled sports activities were shared with their peers and their choice depended on their friends’ participation

*“We (my friends and I) play football in the park” (IP739. Boy, urban environment).*

*“I gave up swimming because none of my friends went” (IP497. Girl, urban environment).*

### 3.3. Mesosystem: Timing and Out-of-School Schedule

**Figure 2:**
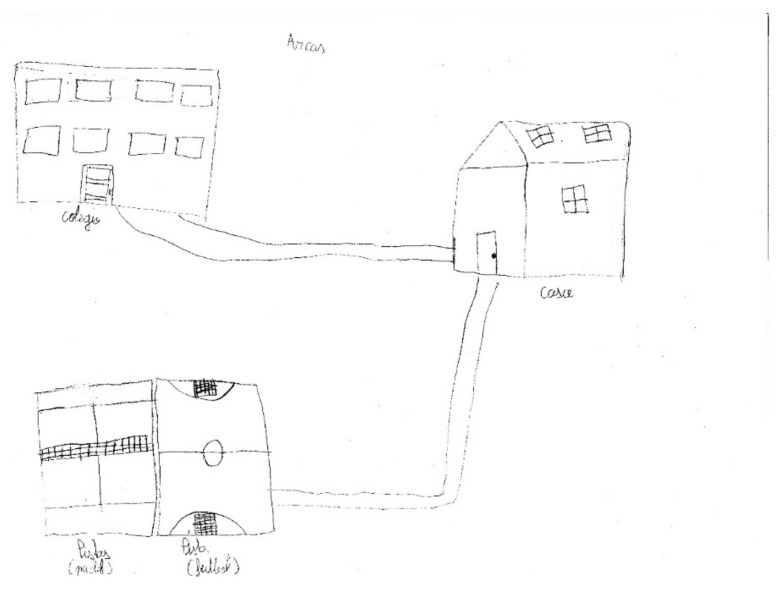
IP722. Boy, rural environment.

**Figure 3:**
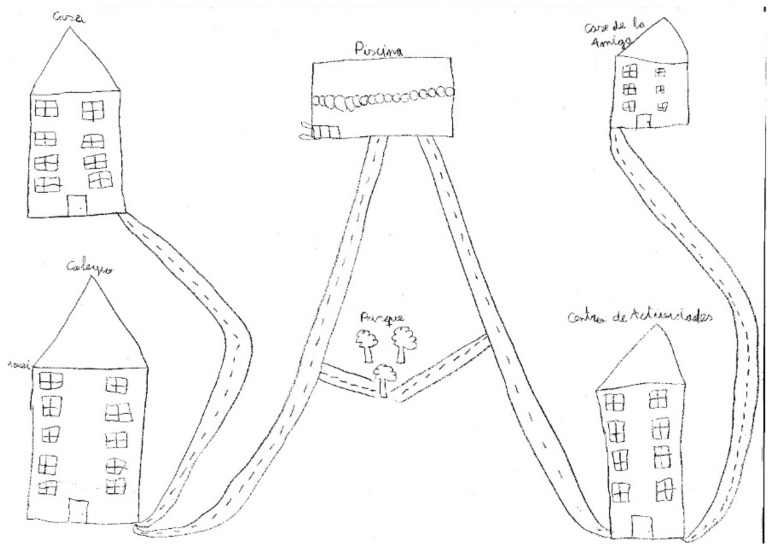
IP499. Girl, urban environment.

From Monday to Friday, the students in urban environments barely had any leisure time and opportunities for spontaneous leisure activities, with these being limited to watching TV or playing videogames. As children started in higher grades, the number and complexity of school tasks also increased, which forced them to give up some of the scheduled activities, especially sports. Therefore, the time dedicated to physical activity and leisure games was reduced. Moreover, the scant offer of extra activities in rural areas limited the participation of children in sport activities, and so they spent more time in the park. The park was a common subject in both urban and rural children’s drawing (e.g., Figure 2 and Figure 3), despite the former reporting they could not go to the park during their leisure time because they had too many scheduled activities.

*“On Mondays (I go to) English lessons, on Tuesdays, violin and then handicraft, on Wednesdays, music and skating, on Thursdays MOVI (physical activity program) and handicraft, and on Fridays music and ballet” (IP634. Girl, urban environment).*

*“On Tuesdays and Thursdays, I watch TV, do my homework, have a snack, watch TV and play Nintendo^®^” (IP734. Boy, urban environment).*

*“Most of the time I spend more time on the computer than watching TV” (IP883. Girl, rural environment).*

*“I played football, went to computer classes and MOVI (physical activity program), but since I came to V (name of village) I can’t anymore“ (IP112. Boy, rural environment).*

*“Interviewer: Where do you go after school?*

*All: To the park!!” (GF22. Mixed group, rural environment).*

*“Girl1: My mum says she is busy with the housework and can’t [come with me].*

*Girl2: My mum says the same” (IP425 y IP426. Girls, rural environment).*

*“I go out with my friends, but not with my dad because he works all day long”(IP670. Boy, rural environment).*

### 3.4. Exosystem: Lack of Safety and Weather

Spontaneous activities with friends were limited due to the lack of safety perceived by parents and by the weather. Thus, we found that younger children were not allowed outside without adult supervision. Moreover, we discovered this supervision was maintained for older girls, but not older boys. In addition, both in rural areas and private residential complexes, where boys and girls lived in proximity and shared common areas, the participants said they spent more time out in the street or in the common areas, respectively, as their parents allowed them to do so.

“*I go to the football pitch to play with some friends” (IP66. Boy, rural environment).*

*“I have lots of friends in the residential area and… we go there with our bikes and all that” (IP734. Boy, urban environment).*

*“Interviewer: Do you go out in the street?”*

*Girl: The thing is I live in C (city center) and our parents don’t let us go out there” (IP719. Girl, urban environment).*

*“Interviewer: Can you go out in the street?*

*Girl: Yes, I can.*

*Boy: Me too, I go to buy bread or to my grandmother’s house” (IP54 and IP.56. Girl and boy, rural environment).*

The practice of physical activity is influenced by weather conditions and daylight hours. Consequently, we learnt that children spent more time in the street or playing more active games in summer and spring. Moreover, the participants claimed to have more leisure time as there was no school and the availability of out-of-school activities was reduced.

*“I play more in the summer because now with activities… I don’t have much time” (IP466. Boy, urban environment).*

*“I use my bike more in the summer” (IP615. Girl, rural environment).*

*“Yes, sometimes during summertime [my mum, dad, brother and myself] go for a ride” (IP700. Boy, rural environment*).

## 4. Discussion

Our results show that individual characteristics (sex and age), microsystem (group of friends and parents), mesosystem (family organization) and exosystem (safety and weather) have an influence on the physical activity performed during leisure time by our 8- to 11-year-old participants. More importantly, the relationships between these factors at different levels reveal that the opportunities for physical activity during leisure time are determined by their schedules. This schedule is negotiated by the family and influenced by the parents’ concerns and necessities (tutoring classes and monitoring) according to the age and gender of their child, and with little regard to the child’s preferences. As a result, leisure is limited to the scheduled academic or sports activity organized by their parents, leaving very little time for leisure physical activity.

There is a consensus on leisure as an opportunity to practice physical activity [10]. However, an increase in sedentary activities during the after-school period among children aged below 18 years is shared by the systematic review by Arundell et al. [60]. The results of this study are consistent with previous studies, showing that most activities performed by children in their leisure time are of a sedentary and educational character [18,36,61,62]. Thus, from Monday to Friday students have a school day that continues beyond school itself, and which is composed of time to do their homework and many scheduled activities, leaving little time for spontaneous leisure activities. In this context, physical activity was generally limited to the participation in scheduled sports activities, whereas non-scheduled activities were mainly non-active ones. As in other studies [30,62,63], our results show that physical activity is limited to the participation of boys and girls in scheduled sport activities.

In spite of the lack of unscheduled leisure time, the park appears to be an important concept in the drawings, on most occasions appearing in the center, despite it not being one of the predominant spaces in their daily routine [64,65]. This might show that both leisure time and the park have an important role for children, as in previous studies [66]. It could also mean that the activities they do during their leisure time are not those that they necessarily like or enjoy, but are those they can do. The choice of activity has been shown to be key to the maintenance of physical activity during adolescence [13,17,31].

Our results show that children perceive their parents want to organize their leisure time schedules. Children remark that parental influence encourages this leisure, as parents usually prioritize academic activities over physical ones when organizing their children’s agenda [32,36], perhaps because they consider they already do enough physical activity at school [67] or because they are not aware of the benefits of physical activity and its consequences on their children’s health. This is an important finding for the promotion of environments that encourage the practice of physical activity in Spain and it requires further research. Previous studies about parenting practices show that less directive styles, characterized by acceptance and involvement practices, are associated with better psychosocial adjustment of children in Spain [13,68,69]. Likewise, they stimulate healthy behaviors and, for instance, support the autonomy of children in increasing their participation in physical activity [13,31,70].

Another significant issue is the lack of safety when children go out. Participants reported that their parents sought activities supervised by a trusted adult in the case of both scheduled and unscheduled activities. Scheduled activities provide this kind of supervision; as shown in other studies, parents usually have problems to balance their working hours, afternoons included, with activities outside home [45,62].

Another significant issue is the perceived lack of safety by parents when children go out. As in previous studies, the lack of safety makes it difficult for children to be active through unscheduled outdoor games, as they are not allowed to go out on their own [27,30,71,72]. Our participants reported that their parents sought activities supervised by a trusted adult in the case of both scheduled and unscheduled activities. Thus, they spend most of their time on indoor sedentary activities, such as playing videogames or watching TV, that are associated with worse body composition, worse physical condition, less self-esteem and socializing behaviors, and poorer learning performance [20,21]. This supervision is less strict as children grow older. Furthermore, children in rural areas have more opportunities for leisure time, but this is limited too. Nevertheless, the perceived lack of safety is paradoxical, as it is not based on the real level of crime, but on perceived insecurity. This is higher when parents assess the risk to their children. Our results were collected in small, low-crime crime towns where lack of safety is percieved by the parents, while children do not agree that parks and playgrounds are unsafe. This contrasts with the results of other studies [73]. An explanation about the tendency towards sedentary activities, especially in the case of girls, could be that, parents usually have problems to balance their working hours, afternoons included, with activities outside home, as shown in other studies [45,62]. However, despite children did not choose these kinds of games, they did not openly complain. It seems they adapt to not playing outdoors accepting their parents’ rules and look for an alternative, such as videogames or waching TV with friends, when possible. Our results contrast with other studies where children are more critical of the their parents’lack of time to play with them [66]. 

Although both boys and girls referred lack of the time and opportunities for physical activity as a barrier, particularly when unscheduled, the results of this study show a greater tendency towards sedentary activities in the case of girls. The girls’ preference for more sedentary activities, such as watching TV, playing videogames or doing handicraft, as the results of previous studies [17,18,19,29,30,36,74]. In this sense, and as has been noted for adolescents, the problem is not only the interest, or the lack of it, of girls towards sport or physical activity, but have fewer opportunities to play outdoors and to engage in non-scheduled physical activity [74,75]. Besides, scheduled ones do not adapt to girls’ likes and preferences [17,18,19,29,36,74]. This situation is more evident in the rural environment, where there are fewer sports activities available for girls than in the urban environment [76]. Likewise, we notice that the tendency towards sedentariness and the lack of opportunities the girls have are powered by a gender socialization that makes sport and physical activity a male pursuit [19,37,74]. This is important due to the influence of early gender socialization and primary agents (family, friends, school) in women’s physical activity participation during childhood, adolescence and middle age [75,77]. 

In Spain, women and girls spending less leisure time and this has been associated with gender inequalities. Despite this, women have more positive leisure experience and time perspectives than men, which stimulates resilience [78]. This occurs when they freely choose activities that, even being little time, satisfy them. Women of any age tend to prefer physical activities that can be performed in a group or outdoors with family [77,78]. Thus, although the socialization of girls is restricted to the leisure time they share with family and friends, girls could have very positive experiences should they had the opportunity to participate in their preferred sports and physical activities.

## 5. Strengths and Limitations

Amongst the strengths of this study, we highlight the use of two qualitative techniques (individual drawings and focus groups). The drawings informed about the main places that participants went to, and helped them to express their opinions and experiences, which provided a more complete and reliable data collection, as well as different levels of depth in the children’s answers to the study questions. Moreover, the use of triangulation methods (data collection, researchers and encoding levels) improved the analysis and the reliability of the results [50,58]. It should be noted that no discrepancies were found between the participants’ drawings and their discourse.

Between its limitations, we should note the age of the participants, from 8 to 11, which increased the complexity of the design and analysis. However, triangulation was used to reduce these limitations [50,58]. Moreover, the points of view in this study belong to the participants in the MOVI2 physical activity only, and may differ from the perceptions of those children not taking part the intervention because they could not have opportunities to join in an after-school sport program, especially in a rural setting. Furthermore, the year in which the study was held, 2011, could make the results out-of-date. But current reviews show that the main studies are focused on parents and adolescents or children in the school environment, being less known the perception of leisure time for 8–11-year-old children [30]. More studies are needed that aim to provide a deeper understanding of children’s preferences.

## 6. Conclusions

To sum up, our results bring to light the fact that the main barrier to leisure time physical activity in children is family, due to the organization of family life. Lack of a balance between work and family life means children have few opportunities to engage in physical activity. Spontaneous, outdoor leisure physical activity is especially limited in urban environments, even though the participants are aware of its importance, as their drawings show. Leisure time is outside the children’s control; their parents manage and establish the kind of activities children do in their leisure time, and education has more importance for them than leisure. Furthermore, the enormous number of scheduled activities keeps children occupied, but inactive, and parents’ perception of a lack of safety puts even greater limits on the opportunities to perform physical activity in younger children and girls of all ages in urban environments.

The results of this study provide key information to improve the interventions aimed at increasing children’s physical activity levels and to help them meet daily physical activity recommendations. In this sense, the findings show that activities promoting physical activity for children should make parents aware of the importance of the physical activity for their children’s healthy development, both physically and mentally. Moreover, it is necessary to enable children to enjoy their outdoor leisure time and choose scheduled sport activities according to their preferences. Thus, children may enjoy their time with friends while also ensuring their motivation to do physical activity. It is also essential to implement policies that provide a variety of good sporting activities in the rural environment, and a gender-equal sport promotion. To conclude, the socio-ecological model must be considered as a comprehensive approach to the factors that influence active play in children. Indeed, this model could be used for designing and evaluating future successful interventions.

## Figures and Tables

**Table 1:** Characteristics of the participants.

	Boys	Girls	Total	
Urban environment	28	36	64	98
Rural environment	8	26	34	
Socio-economic Status	Low		2.5%	
	Middle		85%	
	Upper		12.5%	

**Table 2:** Focus group script.

Main Question	Probing Questions
What kind of activities do you practice in your leisure time?	Do you practice the same activities all days? Do you like them? Are these activities organized? Do you choose the activities?
What kind of games do you play?	Traditional games, sports, electronic devices…
Where do you practice these activities and games?	What are these places like? Green space, recreational space, leisure and sport facilities, weather…
With whom do you practice these activities and games?	Boys, girls, family…

**Table 3:** Barriers and facilitators by level of Bronfenbrenner’s socio-ecological model.

FACTOR	BARRIER	FACILITATOR
INDIVIDUAL CHARACTERISTICS		
Preferences		Children prefer participating in physical activity and games that they enjoy.
Sex	Girls prefer more sociable activities (handicrafts).	Boys prefer more physical activity (football).
Age	Older boys and girls prefer sedentary activities (TV and videogames).	
MICROSYSTEM		
Parents and siblings	Prioritizing educational activities and not physical activities.	Negotiate the activities listening to children’s needs and preferences.
		Parents, especially fathers, are models for participation in physical activity.
	Children do not share physical activity with their mothers.	Children enjoy physical activity with their fathers and siblings.
Friends		Children prefer sharing activities with their friends.
		Children choose physical activities their friends are involved in.
MESOSYSTEM		
Timing	Lack of balance in work and family life, especially for mothers, affects the kind of activities (sedentary) and where (indoor places) children play.	
Schedule	Schedules are full of educational activities with less time for physical activity and leisure games.	
EXOSYSTEM		
Safety	Parents prefer supervised activities, especially for younger children and girls.	
Weather		Daylight hours and good weather conditions facilitate activities in places, such particularly physical activity.
